# A Case of Ophthalmic Branch Trigeminal Neuralgia in the Emergency Department

**DOI:** 10.7759/cureus.3831

**Published:** 2019-01-06

**Authors:** Sebastian R Copp, Constance LeBlanc

**Affiliations:** 1 Emergency Medicine, Dalhousie University, Halifax, CAN

**Keywords:** neurology, trigeminal neuralgia, cephalalgia, headache, pain, tn, ophthalmic nerve, ophthalmic, emergency medicine, family medicine

## Abstract

This case report describes a middle-aged patient with a past history of migraine headaches, who presented to the emergency department with a new onset of headaches around his left eye that were different from the pattern and character of his usual migraine headaches. The pain was severe, brief, and stabbing in nature, lasting only seconds, and occurring over intervals of a few minutes. His vital signs, including glucose, were normal. He had no constitutional symptoms, a normal neurological examination, and a normal head, eyes, ears, nose, and throat examination. The painful paroxysms could not be elicited on palpation of his face, head, or oral mucosa. His blood investigations were reported as within normal limits. He was not using alcohol or any illicit drugs and was not taking any medication. A diagnosis, with supportive imaging, of ophthalmic branch trigeminal neuralgia (TN) was made. His pain responded well to treatment with carbamazepine. TN is characterized by brief and intermittent lancinating pain with or without a constant background level of pain in the sensory distribution of one or more branches of the trigeminal nerve. There are three main causes for TN: idiopathic, the classical type resulting from neurovascular compression, and the secondary type typically due to multiple sclerosis, a space-occupying lesion, or a skull base abnormality. The mandibular and maxillary branches are most affected and can often be affected simultaneously. Ophthalmic branch TN is relatively rare. Virtually all of TN cases are unilateral and most are the classical type. Distinguishing TN from other cephalalgias, ocular pain, dental pain, or other pathology is critical to a proper diagnosis and initiation of effective therapy. Identifying trigger zones is important and carries a high diagnostic yield; however, they may be anatomically difficult to access, or in a refractory period during a physical examination. Physicians should be aware of several red flags associated with a suspected case of TN. Carbamazepine is the first-line treatment for TN, capable of reducing pain in 90% of patients. Failure to respond to medication requires further investigation and/or specialist referral. Untreated or unrecognized TN can have significant impacts on a patient’s quality of life.

## Introduction

A patient presenting with facial pain or a headache carries a potentially expansive differential diagnosis. For example, facial pain can be related to numerous neuralgias (e.g., glossopharyngeal, geniculate), myofascial pain syndrome, Sjogren's syndrome, acute sinusitis, cluster headaches, rhinitis, otitis, glaucoma, among many other conditions. Headaches are one of the most frequent reasons for doctor visits, and the range of causes is vast. Headaches can be caused by dietary factors (e.g., foods rich in dopamine, hydroxyphenylethylamine, or glutamate), substance use (e.g., alcohol), and environmental exposure (e.g., sun). Headaches can result from relatively benign conditions, such as tension headaches, hypotension-induced headaches, or psychosomatic headaches, or more serious and life-threatening conditions such as subarachnoid hemorrhage, meningitis, or a stroke. Headaches can also be caused by numerous infectious, metabolic, toxicological, idiopathic, vascular, otological, dental, and ophthalmological pathologies, further complexifying the identification of the cause of a headache. As with any medical presentation, a detailed history around the character, location, onset, radiation, intensity, timeline, frequency, and provoking and palliative factors of the pain and associated symptoms, as well as a relevant personal and family medical history can significantly narrow the differential diagnosis. Some conditions that cause headaches can be almost pinpointed through key details obtained in a thorough history and physical examination. One such condition is trigeminal neuralgia (TN).

TN, also known as "tic douloureux," is a relatively uncommon condition with an annual incidence in the general population of 4.3 per 100,000. Incidence increases with age, peaking between the fifth and eighth decades of life, and can be as high as 1 per 1000 in patients over the age of 75. There is also a female preponderance of about 2:1 [1]. There have been reports of TN being more prevalent in some families [2]. TN is characterized by a very sharp, intense, lightning-like lancinating pain, which occurs in one or more divisions of the trigeminal nerve, almost always unilaterally, in bursts lasting seconds but that can last up to a few minutes. These paroxysms occur over a longer time frame constituting what is referred to as a "painful period." TN can have periods of complete remission from the paroxysmal episodes, with about 63% of patients having remission periods ranging from weeks to years [3]. The length of painful periods and periods of remission are variable. During painful periods, patients can have anywhere from three to 70 episodes per day [2].

TN can also present with an aching, burning, throbbing, lower-intensity pain, which can persist in between episodes or even precede the development of paroxysms, which is known as TN with concomitant continuous pain, atypical TN, or TN type [4-5]. "Typical" TN is characterized by a greater than 50% episodic pain, whereas atypical TN is generally characterized by a greater than 50% constant/background pain. This helps to illustrate that TN can present as a broad continuum between purely paroxysmal pain, and paroxysmal pain with a constant background of pain [6]. Atypical-type TN is reportedly more common than typical TN [4]. Conveniently, the first-line treatment for typical and atypical TN is the same [7].

## Case presentation

A 45-year-old man presented to the emergency department (ED) complaining of a headache around his left eye. This had started 48 hours prior to consulting, and he explained that he had had migraine headaches that were different than this pain, in both their pattern and character of pain. He had not slept since the onset of the pain. He described this headache as a recurrent brief and severe lancinating pain, lasting only seconds, and occurring at three- to five-minute intervals. Each episode of pain, although brief, was so severe that he was unable to function with his symptoms. He had complete resolution of pain between episodes with no background symptoms. There were no other associated symptoms such as fever, chills, nausea, or vomiting. During or between paroxysms, he did not have autonomic symptoms or any other neurological symptoms, and he had not noticed any exacerbating or alleviating factors. He reported no shoulder girdle stiffness or general malaise, his appetite was preserved, and he had not had jaw claudication. The patient had not had any type of trauma. There was no reported alcohol or illicit drug use. The patient had a past medical history positive for migraines, and he was not taking any prescribed or over-the-counter medications.

At triage, the physical assessment revealed normal vital signs, visual acuity, glucose, and mentation. On physical examination, he was not distressed at baseline, however, during the episodes, his pain caused him significant distress. The examination of his eye was normal with no conjunctival injection. His cranial nerve examination was normal, as well as his peripheral neurological examination. The examination of his ears, nose, and throat was normal. No headache trigger zones were located around his mouth, ears, eyes, or scalp. He did not have tenderness in the temporal region on palpation, and temporal pulsations were normal. The skin on his face and head was intact without erythema, blistering, altered sensation, or tenderness. Routine blood work (complete blood count (CBC), glucose, electrolytes, blood urea nitrogen (BUN), creatinine, erythrocyte sedimentation rate (ESR)) was within normal limits. No imaging was obtained at this visit.

Due to the character and pattern of this pain, a presumptive diagnosis of ophthalmic branch TN was made, and treatment with carbamazepine was initiated. An outpatient ophthalmology appointment was arranged, and he was discharged home with instructions to return to the ED if he were to develop a fever, worsening headache, neurological symptoms, or a rash. At his follow-up visit with the ophthalmologist 72 hours later, he remained symptomatic, however, he has been able to sleep and function better since having initiated treatment. An unenhanced CT scan of the head was obtained, which was normal. No further testing was deemed necessary, and a diagnosis of ophthalmic branch TN was confirmed. Symptoms continued to resolve over the next week with ongoing treatment with carbamazepine.

## Discussion

According to the International Classification of Headache Disorders (ICHD-3), there are three broad classes of TN: classical TN, TN due to a secondary cause, and idiopathic TN. TN is a clinical diagnosis, but the three types have different causes, which can be differentiated with imaging [3,8]. Classical TN is due to neurovascular compression of the nerve branches as they leave the brainstem, resulting in focal demyelination, which can be demonstrated with MRI [2,9]. Secondary TN is due to either multiple sclerosis lesions causing the symptoms, a space-occupying lesion, or other less common causes, such as a skull base abnormality. Secondary TN constitutes about 15% of cases of TN [3]. Idiopathic TN has no identifiable cause. The clear majority of cases (80%-90%) of TN are the classical type, which should not carry sensory deficits [3,5,10].

Classical and idiopathic TN present virtually always as unilateral painful paroxysms. The right side of the face is affected 64% of the time [1]. Of the trigeminal nerve divisions, the most commonly affected is the mandibular division (55%) followed by the maxillary division (39%). TN in either of these divisions may be misinterpreted as originating from alveolar or dental pain. The ophthalmic branch is rarely affected, and the pain can be interpreted as coming from the nose, eyes, or head around the area of the scalp [1,3]. "Trigger points/zones" are a discerning feature of TN whereby episodes can be triggered by cutaneous stimulation or movement of a specific region of the face served by the affected division of the trigeminal nerve. It has been noted that trigger zones can be rendered inactive for a "refractory" period following a painful paroxysm, lasting anywhere from seconds to minutes [3-5,11].

True bilateral classical or idiopathic TN in which both sides are simultaneously affected is exceedingly rare. Simultaneous involvement of both sides of the face would suggest TN due to another condition, such as multiple sclerosis. However, it has been observed that both the nerve branch affected and the side of the face can change over time. It is, therefore, important to clarify the timeline of patient-reported bilateral TN-like pain, specifically if both sides were ever simultaneously affected [3,5].

Reportedly, the most common presentation of TN is an intra-oral toothache-like pain with atypical qualities (continuous aching, burning). It is important to distinguish alveolar dental pain from TN in any case where the pain is deemed to originate around the teeth. In these cases, trigger points may be localizable intra-orally [4]. Identifying trigger zones are helpful in securing a diagnosis of TN. Recall that trigger zones may either be refractory or located in an inaccessible anatomical location. Pain from activating trigger zones, or during paroxysms, must not radiate beyond the affected trigeminal nerve branch’s distribution. If so, consider other potential items in your differential diagnosis [5]. Trigger zones activated either by cutaneous/mucosal stimulation, or movement, have been reported in 99% of cases of classical TN and, therefore, serves a high diagnostic utility in this category [3]. The trigger zone may be in a location different than where the pain is felt to originate from and activating a trigger zone will cause pain to radiate through the area served by only by that division of the trigeminal nerve. A variety of stimuli may activate a trigger zone: a puff of air, light or heavy touch, or mechanical stimulation from a muscular movement in the face or head [3].

It is important to differentiate TN from the presentation of trigeminal autonomic cephalalgias (TACs). TN and TACs can appear very similar, but they are primarily differentiated by the presence and degree of autonomic symptoms during painful episodes. Autonomic features must be present for a diagnosis of a TAC. While TN is not characterized by autonomic symptoms, mild symptoms may occur during paroxysms (e.g. lacrimation or redness of the ipsilateral eye) [5]. TAC pain is usually longer in duration than TN during a paroxysm, and the patient will more commonly appear restless or agitated [5]. Treatment of TACs differs from that of TN, making this distinction clinically important [12].

When a patient with previous migraine headaches presents with a headache described as “new or different,” a careful history and physical examination are indicated to explore other potential causes of this headache presentation. A neurological assessment, including cranial nerves and a cerebellar and peripheral examination, is indicated. A head and neck examination are required, including the skin and the mucous membranes. If there are any cranial nerve abnormalities, imaging and/or specialist consultation are indicated [2]. Vital signs and blood glucose must be measured. Follow-up with the family physician, and instruction to return to the emergency department if a rash, fever, worsening headache, or new neurological symptoms develop, is a prudent plan when discharging patients.

There are red flags associated with a presentation of TN that may suggest a more onerous cause of the symptoms. Careful focus during history and physical exam should be paid to: neurological or sensory deficits, abnormal trigeminal reflexes, difficulty achieving pain control or poor response to carbamazepine, history or presence of any skin lesions or oral lesions with the potential of perineural spread, ophthalmic division-only TN, simultaneous bilateral TN (suggestive of benign or malignant lesions or multiple sclerosis), age of onset under 40 years of age, optic neuritis, or a family history of multiple sclerosis [2-3,7]. There is currently debate in the literature as to whether ophthalmic division-only TN is a veritable red flag [3]. According to the ICHD-3, hypoesthesia and/or hypoalgesia in the affected trigeminal nerve branch would indicate axonal damage and trigeminal neuropathy, requiring a more detailed workup [5].

Classical TN is principally a clinical diagnosis [3,7]. While MR imaging showing neurovascular compression is technically needed for a diagnosis of classical TN, the current American Academy of Neurology (AAN) guidelines suggest there is insufficient evidence to recommend for or against the use of MRI to detect neurovascular compression associated with classical TN [5,7]. Based on the best available evidence, in the absence of abnormal trigeminal reflexes, sensory deficits, and bilateral involvement, it is considered permissible to forgo obtaining MR imaging of the head [7]. The study informing the AAN guidelines suggests there is weak evidence for considering the use of routine head imaging in patients with TN, in that it can identify a cause in 15% of cases [7]. It is important to note that bedside testing of the sensation of the trigeminal nerve branches has been reported to lack the sensitivity of a more refined test (i.e., quantitative sensory testing) to detect true sensory deficits [3]. Similarly, the AAN guidelines suggest there is good evidence to test trigeminal reflexes in a qualified electrophysiological laboratory, which may help elicit abnormalities not detected on physical examination. Either of these findings would suggest secondary TN and would indicate head imaging [3,7]. Consider that secondary TN may still present with unilateral involvement and intact sensation [7].

Carbamazepine and oxcarbazepine are the first-line treatment for TN. Carbamazepine is effective in reducing pain for 90% of TN cases, with 70% of patients achieving a full response in pain reduction [2-3]. It is recommended to begin treatment with a low/minimal dose and increase the dose every three to seven days to optimize pain control with minimal side effects [2]. Ancillary evaluation prior to anticonvulsant therapy may be indicated for some patients. These include, but are not limited to, liver function parameters and subsequent monitoring of serum anticonvulsant levels. If TN is non-responsive to anticonvulsant therapy, referral for imaging and/or consultant assessment are indicated [2]. This may involve MR imaging of the brainstem to look for signs of neurovascular compression that may be treatable with neurosurgery. MRI has a reported sensitivity of 96.7% and a specificity of 100% for identifying the neurovascular compression of a trigeminal nerve branch [8]. However, without specific imaging techniques and 3D reconstruction, the specificity of MRI detection of neurovascular compression can be quite limited [3].

If a patient’s life is being severely affected by TN, consider referral to a patient support group or interdisciplinary management among other healthcare professionals. TN has been shown to be associated with depression, increased risk of suicide, and anxiety from a fear of developing another painful paroxysm [2].

## Conclusions

When a patient with a history of cephalalgia presents with a change in its clinical features or neurological abnormalities on clinical evaluation, a thorough assessment is indicated. Although ophthalmic branch TN is rare, consider it in patients with paroxysmal pain in or around the eye and scalp. Collect a history, with specific attention to the presence of autonomic symptoms, location and radiation, type and intensity of pain, frequency and time course of the episodes, and red flags. Localizable trigger points will contribute positively to, but are not required for, a diagnosis of TN. Imaging is likely required if there are abnormalities with trigeminal nerve sensation or reflexes or if there is bilateral involvement. Carbamazepine is the first-line treatment for classical and idiopathic TN and will be effective in most cases. If symptoms persist despite medical treatment with a decreased quality of life and functionality, imaging, and/or consultation to neurology or neurosurgery may be indicated.
